# Tumor-penetrating peptide boosts bispecific T-cell engager antitumor efficacy for the pancreatic cancer

**DOI:** 10.3389/fimmu.2025.1693755

**Published:** 2025-12-04

**Authors:** Lu Zou, Jingjing Chen, Xinyuan Bai, Yingxin Wang, Changchang Lu, Qiaoli Wang, Subiyinuer Tuerhong, Mengzhu Li, Qinghua Zheng, Fanyan Meng, Juan Du

**Affiliations:** 1 Department of Oncology, Nanjing Drum Tower Hospital, Clinical College of Nanjing Drum Tower Hospital, Nanjing University of Chinese Medicine, Nanjing, China; 2 The Comprehensive Cancer Center of Nanjing Drum Tower Hospital, Affiliated Hospital of Medical School, Nanjing University, Nanjing, China; 3 Department of Laboratory Medicine, Nanjing Drum Tower Hospital, Clinical College of Nanjing Medical University, Nanjing, China; 4 Institute of Translational Medicine, Zhejiang University, Hangzhou, China; 5 State Key Laboratory of Analytical Chemistry for Life Sciences, School of Chemistry and Chemical Engineering, Nanjing University, Nanjing, China

**Keywords:** KRAS G12V, bispecific T-cell engager (BiTE), IRGD, pancreatic cancer, T-cell infiltration

## Abstract

**Background:**

One of the main hurdles in solid tumors to the limited response of immunotherapy is the lack of sufficient T-cell infiltrate. This study aims to construct an iRGD-modified BiTE-directed T-cell therapeutic approach to enhance the treatment efficacy against KRAS G12V-mutated pancreatic cancer.

**Methods:**

We used a novel bispecific T-cell engager (BiTE) targeting the HLA-A2/KRAS G12V complex and CD3 (HLA-A2/KRAS G12V-CD3 BiTE). By modifying with iRGD, we induced BiTE-mediated inward flow of activated effector T cells, specifically targeting the KRAS G12V mutation and improving tumor tissue penetration to address the problem of limited efficacy due to insufficient effector cells infiltration.

**Results:**

The results demonstrated that iRGD modification could promote tumor-specific lymphocyte infiltration and accumulation in tumor tissue, significantly inhibit tumor growth, and prolong survival in a xenograft pancreatic tumor model. This dual-action approach enhances T-cell infiltration by promoting transvascular and stromal penetration, greatly enhancing the efficacy of bispecific antibodies in solid tumors, leading to effective tumor eradication.

**Conclusions:**

These findings strongly suggest further clinical validation of this iRGD-modified BiTE-directed T-cell therapeutic approach, potentially offering a more effective treatment option for patients with pancreatic cancer and other solid tumors.

## 1 Introduction

Pancreatic cancer is a highly malignant gastrointestinal cancer with limited therapeutic options and a 5-year survival rate of less than 10% (1). In the case of pancreatic cancer, 90% harbor mutations in the oncogene KRAS (2). G12V mutation accounts for approximately 30% (3), which is an attractive therapeutic target. However, the development of drugs that directly target KRAS mutations faces many challenges. Since it is a very common genetic variation, which makes the development of targeted drugs for this mutation particularly important.

Immunotherapy using bispecific antibody (bsAb) are emerging as a promising approach that improves the outcome of solid tumors treatment. BiTE is a bispecific antibody formed by linking the single-chain variable fragments (scFvs) of two antibodies that binds surface antigens on tumor cells and CD3ε on T cells (4). Upon binding to its specific targets, a BiTE molecule forms a bridge between T cells and tumor cells. This structure and specificity enable the BiTE to physically link T cells to tumor cells, thereby stimulating T cell activation and proliferation, cytokine production, target cancer cells lysis (5, 6). Tarlatamab, DLL3-targeted bispecific T-cell engager (7), received approval from the U.S. Food and Drug Administration (FDA) in 2024 for clinical application in the treatment of patients with extensive-stage small-cell lung cancer (ES-SCLC) who experienced disease progression during or following platinum-based chemotherapy. In Phase I/II clinical trials, tarlatamab exhibited sustained antitumor effects and demonstrated a favorable safety profile with manageable side effects (8, 9). Although the success of tarlatamab has offered hope for oncological treatments, significant challenges remain in the realm of immunotherapy, particularly for ‘cold’ tumors (10). The most prominent characteristic of ‘cold’ tumors is poorly infiltrated with T cells (11). Insufficient numbers of T cells in the tumor microenvironment, response rates to immunotherapeutic strategies are significantly diminished. Therefore, enhancing T cell infiltration is essential in enhancing the efficacy of immunotherapies in solid tumors.

Since 2009, Sugahara et al. reported iRGD as a cyclic peptide with tumor-specific penetrating capacity in their study published in Cancer Cell and Science (12, 13). The tumor-penetrating peptide iRGD (CRGDKGPDC) mediates potent tumor-specific delivery through a sequential, dual-receptor mechanism. The process initiates when the RGD motif of iRGD binds to αvβ3/β5 integrins on tumor endothelium and tumor cells. Following this initial docking, the peptide is proteolytically cleaved, exposing a C-terminal CendR motif. This activated CendR motif then binds with high affinity to neuropilin-1 (NRP-1), triggering CendR-mediated transvascular transport and transient stromal permeability. This orchestrated cascade not only promotes the deep penetration of the iRGD-coupled agent itself but also facilitates the co-transport of co-administered therapeutics and immune cells into the tumor tissue via “bystander effect”.

**Figure 1:**
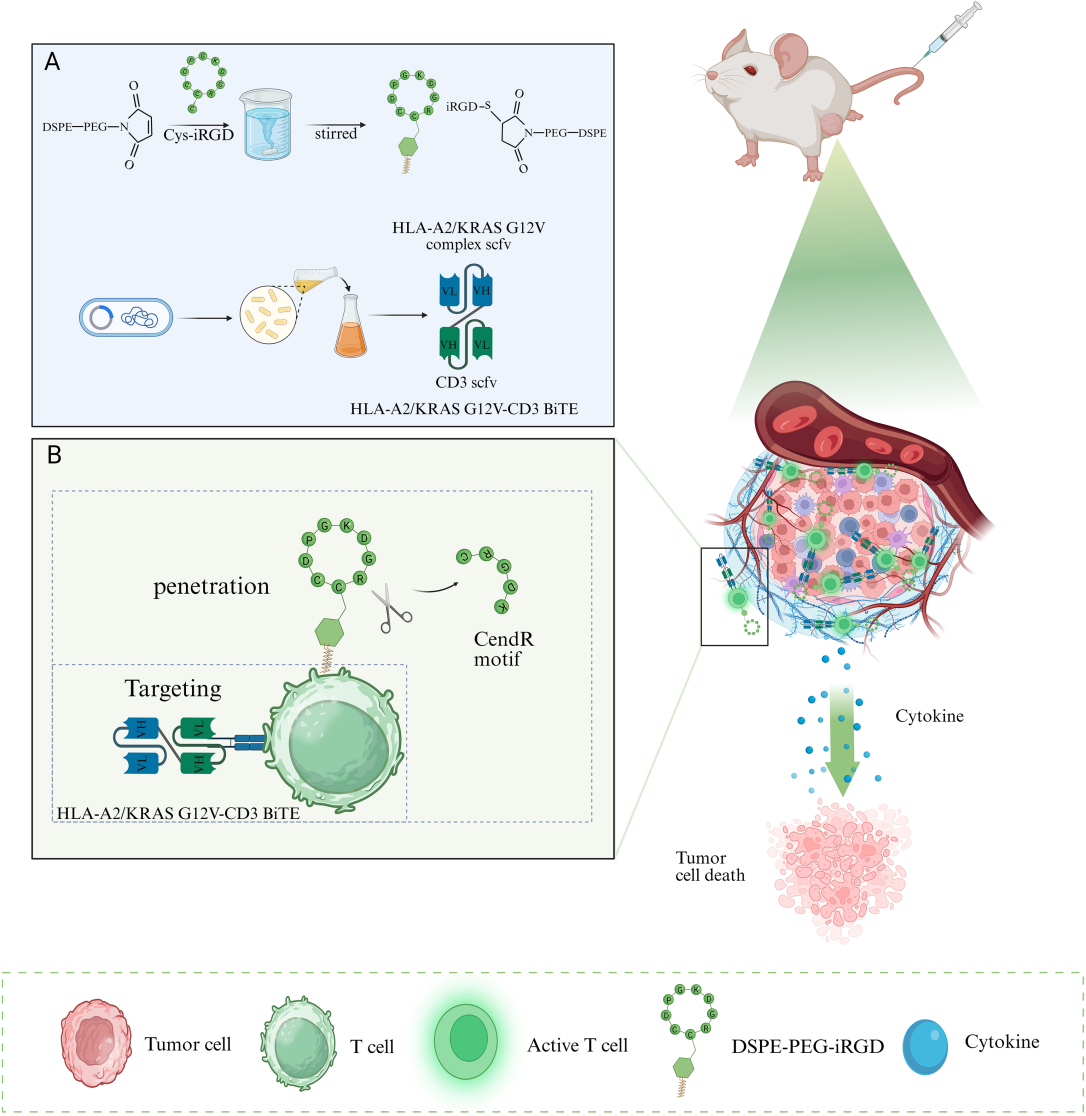
The design of iRGD-modified BiTE-directed T-cell therapeutic approach and the immunotherapeutic mechanism involved. Created with BioRender.com. **(A)** Structure of DSPE-PEG-iRGD and the bispecific T-cell engager (BiTE). **(B)** Two synergistic functions: (i) Penetration: Mediated by the iRGD peptide, which promotes deep tissue infiltration of T cells. (ii) Targeting: Achieved by the BiTE, which binds the HLA-A2/KRAS G12V complex on tumor cells and CD3 on T cells.

Based on these, we engineered the surface of bispecific antibody-modified T cells with iRGD would confer dual functionality: the established targeting capability afforded by the bispecific antibody, directing T cells to tumor-specific antigens, would be synergistically combined with enhanced penetration driven by iRGD’s sequential binding to αv integrins and subsequently to NRP-1. The combined strategy would promote more robust T-cell infiltration into the tumor parenchyma, enabling efficient execution of their cytotoxic effects (**Figure 1**). Herein, we used a BiTE targeting HLA-A2/KRAS G12V complex and CD3 and assessed the impact of iRGD modification on the capacity of BiTE to enhance T cell activation. Meanwhile we tested the penetration and cytotoxicity of the dual-modified T in the multicellular spheroids (MCSs). We also validated its effectiveness in a typical cold tumor, a xenograft pancreatic tumor model. We constructed a BiTE-directed T-cell therapeutic approach via tumor-penetrating peptide modification that addresses targeting and infiltration issues in tumor therapy. This approach offers the potential for bsAb-based immunotherapy of solid tumors.

## 2 Materials and methods

### 2.1 Cell lines and reagents

All human pancreatic cancer cell lines CFPAC-1 and Capan-1, the gastric cancer cell line NUGC-4 were cultured in Dulbecco’s Modified Eagle Medium (DMEM) and Roswell Park Memorial Institute (RPMI) 1640 medium, respectively. All cultures were supplemented with 10% FBS at 37 °C with 5% CO2 in a humidified incubator.

The cyclic peptide Ac-CCRGDKGPDC-NH2 (C-iRGD) containing an extra cysteine at the C-terminus of iRGD, as well as C-iRGD with FAM conjugated at the N-terminus were purchased from Top-Peptide Ltd. (Shanghai, China). 1,2-Distearoyl-sn-Glycero-3-Phosphoethanolamine-N- [Maleimide (polyethylene-Glycol)-3400] (DSPE-PEG-MAL) was purchased from Laysan Bio, Inc (Arab, AL, USA).

### 2.2 Isolation and culture of human T cells

Peripheral blood mononuclear cells (PBMCs) were obtained from healthy donor blood and isolated using Ficoll density gradient centrifugation. For T cell activation, PBMCs were cultured in AIM-V medium (Gibco, USA) for 4–6 hour for adherence. Non-adherent T lymphocytes were activated by adding 50 ng/mL OKT3 (eBioscience, USA) to the medium on day 1. Activated T cells were expanded in AIM-V medium containing 10% FBS, 300 U/mL IL-2 (Peprotech, USA), 50 ng/mL IL-7 (PeproTech, USA), and 50 ng/mL IL-15 (PeproTech, USA).

### 2.3 HLA-A2/KRAS G12V-CD3 BiTE preparation and characterization

HLA-A2/KRAS G12V-CD3 BiTE was prepared as previously reported (14). Briefly, the protein was expressed in Escherichia coli (E. coli) BL21 (DE3) and induced by isopropyl β-D-1-thiogalactopyranoside (IPTG). The bacteria were harvested and disrupted by sonication. The inclusion bodies were dissolved and subsequently dialyzed in urea. The production was further purified using the His Trap HP column (GE Healthcare, CT, USA) through the SDA-100 system (CELLPRO, Suzhou, China) according to the manufacturer’s instructions. The eluted solutions were dialyzed against PBS, and confirmed by 12% sodium dodecyl sulfate-polyacrylamide gel electrophoresis (SDS-PAGE) analysis and western blot (WB) analysis using an anti-His antibody (Abcam, ab5000). After that, HLA-A2/KRAS G12V-CD3 BiTE concentration was determined with the BCA assay kit (NCM Biotech, Suzhou, China), sterile-filtered (0.22 µm), and stored at -80°C.

### 2.4 Synthesis of DSPE-PEG−iRGD

As previously described (15), DSPE-PEG-Mal and C-iRGD or C-iRGD with FAM were mixed at a 1:1 molar ratio in Hepes buffer (pH = 6.5) and allowed to react at room temperature for 48 h under nitrogen gas. The reaction mixture was then dialyzed in deionized water for 48 h to remove the free iRGD, and the resulting solution was lyophilized.

### 2.5 Dual modification of T cells

The activities T cells were cultured in the presence of IL-2、IL-7、IL-15 for 14 days. Subsequently, T cells were incubated with 10 µg/mL HLA-A2/KRAS G12V-CD3 BiTE for 60 min at room temperature as previously described (14). In order to investigate the parameter of surface modification, T cells modified with HLA-A2/KRAS G12V-CD3 BiTE were mixed with different concentrations of FAM labeled DSPE-PEG-iRGD followed by an incubation period of 16 h at 37 °C and 5% CO_2_. The cells were collected by centrifugation and analyzed using a Cytoflex flow cytometry (Beckman Coulter, USA).

Cell morphology was observed using an optical microscope post BiTE and DSPE-PEG iRGD dual modification. To assess the stability of DSPE-PEG-iRGD modified in the cell surfaces, dual-modified T cells were incubated at saturation quantity, the cells were analyzed by flow cytometry (Beckman Coulter, Brea, CA, USA) at indicated time intervals and the data was analyzed using Flowjo V.10.4 software.

### 2.6 Flow cytometry analysis

To evaluate BiTE binding, 1×10^5^ tumor cells or T cells were respectively co-incubated with BiTE for 6 h at room temperature, followed by PBS washing, and then incubated with anti-His tag antibody (Biolegend, USA) at 4 °C for 30 min in the dark. Subsequently, the cells were washed and examined using flow cytometry.

To examine T cell activation, T cells cocultured with CFPAC-1 and Capan-1 tumor cells at an effector-to-target (E:T) ratio of 10:1 with HLA-A2/KRAS G12V-CD3 BiTE and DSPE-PEG-iRGD were incubated in 96-well plate for 24 h at 37 °C with 5% CO_2_. The supernatant fluids and cells were then harvested and stained with a fluorescently-labeled mouse anti-human antibody followed by a 30 min incubation at 4 °C in the dark. The CBA Human IFN-γ Flex Set (BD Bioscience, USA) and the CBA Human IL-2 Flex Set (BD Bioscience, USA) was used for the secretion of interferon-γ and IL-2. For phenotypic T cell characterization, the following antibody were employed: anti-CD3-PE (Beckman Coulter, USA), anti-CD4-APC (Beckman Coulter, USA), anti-CD8-FITC (RPA-T8, BD Bioscience), anti-CD69-PC5 (Beckman Coulter, USA), and anti-CD25-APC-700 (Beckman Coulter, USA). Flow cytometry was used to conduct fluorescent expression analysis.

### 2.7 Cytotoxicity assays

CFPAC-1 and Capan-1 cells were initially labeled with Carboxyfluorescein succinimidyl ester (CFSE) (Invitrogen, USA) for 10 min in PBS. Following labeling, the dual-modified T cells were added into the tumor cells at various effector-to-target ratios in a 48-well plate. The mixed cells were left to incubate for 48 h at 37 °C with 5% CO_2_ and then Propidium iodide (PI) (Sigma, USA) was added for 10 min in the dark to mark tumor cell death. Subsequently, the cells were analyzed using flow cytometry.

The vitality of dual modified T cells was also assessed by using a carboxy fluorescein succinimidyl amino ester and propidium iodide (CFSE/PI) assay.

### 2.8 The effect of T cells on multicellular spheroids

Capan-1 cells were added in a 96-well plate (round bottom, ultra-low attachment surface, Corning, USA) containing DMEM medium supplemented with 10% FBS (300 cells/well). MCSs were observed under a light microscope and the uniform and compact tumor spheroids were selected for the subsequent studies when their size reached 200-300 µm. When culturing with T cells, HLA-A2/KRAS G12V-CD3 BiTE modified T cells, dual-modified T cells for 16 hours (E/T = 10:1), the MCSs were washed and incubated in fresh DMEM medium. The diameters of the spheroids were monitored.

To study the MCSs penetration of T lymphocytes *in vitro*, T cells were initially labeled with CFSE for 10 min at 37 °C in PBS. Different groups of CFSE-labed T cells were then added to 96-well plate (E/T = 10:1). After incubation for 16 hours at 37°C with 5% CO_2_, the MCSs were imaged using optical microscopy (Leica, Germany). Images were acquired at the midheight of the spheroids and surface plots were generated using Image J software.

For cytotoxicity assay in MCSs, different group of T cells co-cultured with MCSs. After incubation 16 h, MCSs were washed and stained with Calcein AM and PI solutions (Viability/Cytotoxicity Assay Kit, Absin, China) as per the manufacturer’s instructions. The images were then processed using ImageJ software to quantification live/dead cells.

### 2.9 Tumor inoculation and treatment in BALB/c nude mice

In a human pancreatic cancer tumor model, An equal mixture of 3×10^6^ CFPAC-1 tumor cells and Matrigel (BD Biosciences) were injected (s.c.) on the right lower sides of the abdomen of each BALB/c nude mouse (female, five weeks old, weighing 14–16 g) for subcutaneous tumor effect experiments. The subcutaneous tumor-bearing mice were randomized into four groups of different treatment (n=6) (1). NS (2) T (3) BiTE-T (4) BiTE-T-iRGD when the tumor volume reached 80~100 mm^3^. Mice received intravenous administrations of NS (100 µl), HLA-A2/KRAS G12V-CD3 BiTE (100 µg), T cells (1×10^7^), DSPE-PEG-iRGD (200 µg). Tumor volume and body weight were monitored every 2 days, and tumor volume was calculated using the formula length×width^2^×0.5. Tumor dimensions were measured using a digital caliper. The mice were humanely euthanized when the tumor volume reached ethical human endpoints, exceeded 1500 mm^3^.

### 2.10 Biosafety evaluations

For safety studies, three mice from each group were randomly selected and the main organs (Heart, Liver, Spleen, Lungs, and Kidneys) were harvested, fixed in 4% paraformaldehyde, sectioned, and stained with H&E for histology analysis two weeks after the start of treatment. Alongside collecting retro-orbital blood to measure biochemical markers (ALT, AST, CREA, UREA) to assess systemic toxicity. Histology analysis was performed using optical microscopy (DM5000, Leica, Germany).

### 2.11 Biodistribution and immunofluorescence confocal imaging

To evaluate the tumor targeting efficiency of T cells modified with HLA-A2/KRAS G12V-CD3 BiTE and DSPE-PEG-iRGD in tumor-bearing mice, 10^7^ T cells of different groups labeled with near-infrared fluorescent probe DiR (MedChemExpress, MCE, China) were injected intravenously in CFPAC-1 subcutaneous tumor model. The tumors and organs were analyzed using the CRi Maestro™ Imaging System (Cambridge Research Instrumentation) at the different time points following T-cell reinfusion. At the end of the observation time point, tumors and organs, including heart, liver, spleen, lung, and kidney, were excised and imaged from one mouse in each group.

To investigate the penetration of T cells *in vivo*, different groups of CFSE-labed T cells were injected intravenously tumor-bearing mice. The mice were sacrificed and the tumors were harvested 48 h after administration. Immunofluorescence of the frozen tumor tissue sections were mounted with DAPI (Beyotime, Shanghai, China).

### 2.12 Ethical statement

All animal procedures were conducted in accordance with the guidelines set by the Animal Care Committee at Drum Tower Hospital (Nanjing, China) under the institutional approval number 2024AE01061. The Ethics Committee of Drum Tower Hospital approved all experiments in this study.

### 2.13 Statistical analysis

Statistical analysis was completed through GraphPad Prism V.9.0.0 (GraphPad Software, San Diego, CA, USA). All results are presented as means ± SEM. Statistical significance between different experimental groups was analyzed using student’s t test or two-way ANOVA. A P value of less than 0.05 was considered significant. (ns p > 0.05, *p < 0.05, **p < 0.01, ***p < 0.001 and **** p < 0.0001).

## 3 Results

### 3.1 Generation and characterization of HLA-A2/KRAS G12V-CD3 BiTE

**Figure 2:**
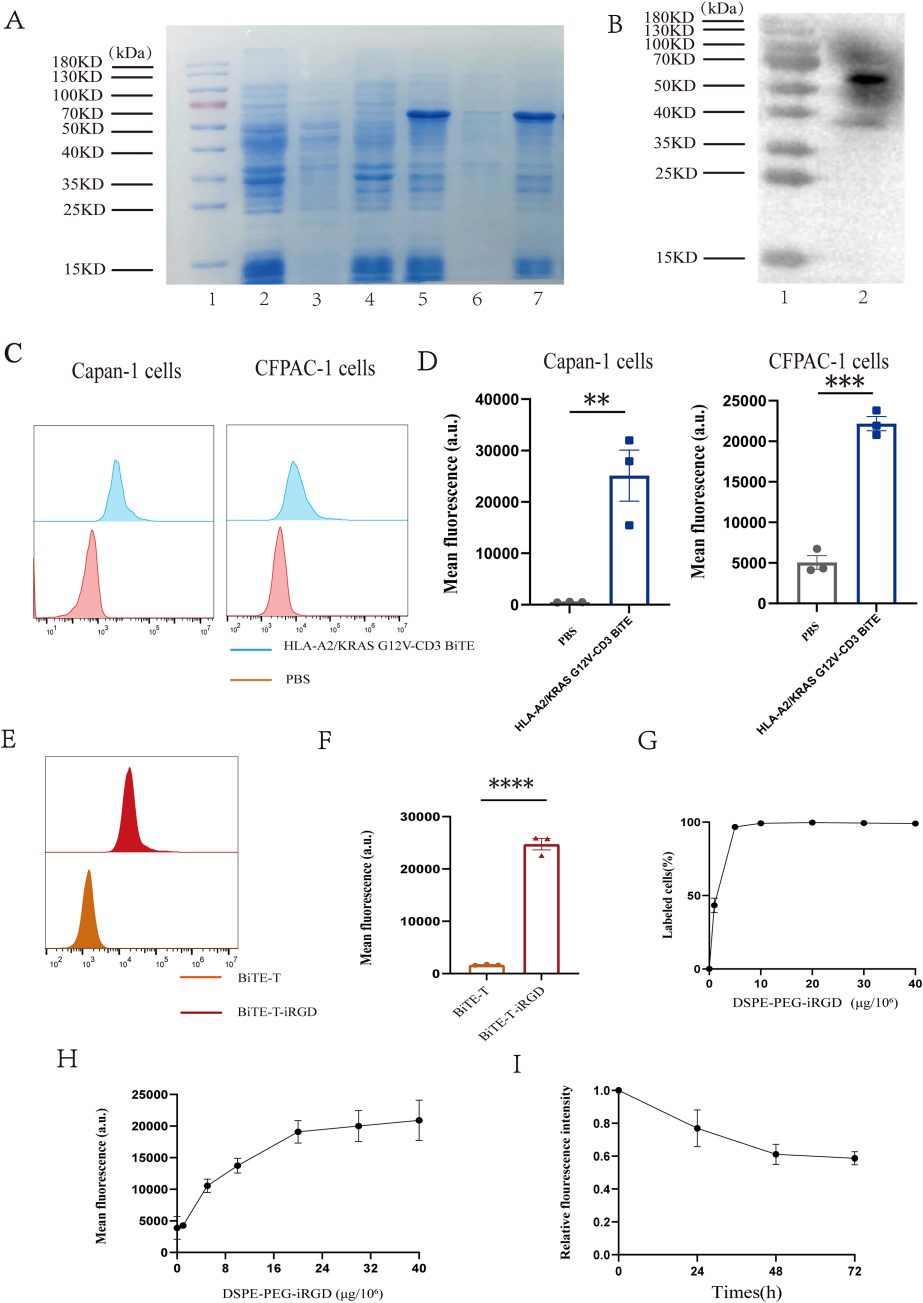
Construction of HLA-A2/KRAS G12V-CD3 BiTE and DSPE-PEG-iRGD. **(A)** HLA-A2/KRAS G12V-CD3 BiTE is induced and expressed as inclusion body. Lane 1, marker; lane 2, bacterial cultures without IPTG; lane 3, supernatant of bacterial cultures without IPTG; lane 4, inclusion bodies without IPTG; lane 5, bacterial cultures with IPTG; lane 6, supernatant of bacterial cultures with IPTG; lane 7, inclusion bodies with IPTG. **(B)** Eluted fractions were identified by western blot analysis. Lane 1, marker; lane 2, purified HLA-A2/KRAS G12V-CD3. **(C)** Flow cytometry results showing bindings of HLA-A2/KRAS G12V-CD3 BiTE to Capan-1 and CFPAC-1. **(D)** Mean fluorescence intensity on Capan-1 and CFPAC-1 in **(C)**. **(E)** Flow cytometry histograms of BiTE-T cells and the cells incubated with DSPE-PEG-iRGD-FAM. **(F)** Mean fluorescence intensity in **(E)**. **(G)** Analysis of the percentage of DSPE-PEG-iRGD-FAM modified BiTE-T cells using flow cytometry. **(H)** Mean fluorescence intensity of BiTE-T cells with different dosage of DSPE-PEG-iRGD-FAM in **(G)**. **(I)** Flow cytometric analysis of changes in relative averaged fluorescence intensities of BiTE-T cells modified with DSPE-PEG-iRGD-FAM over the culture periods. Data are represented as mean ± s.e.m.; n = 3. **p < 0.01, ***p < 0.001, ****p < 0.0001, ns, not significant. BiTE-T; T cells modified with HLA-A2/KRAS G12V-CD3 BiTE; BiTE-T-iRGD, BiTE-T cells modified with DSPE-PEG-iRGD.

HLA-A2/KRAS G12V-CD3 BiTE was generated by scFv of the HLA-A2/KRAS G12V complex and the scFv of the anti-CD3 antibody. The resulting protein was successfully induced in E. coli BL21. SDS-PAGE analysis under reducing conditions revealed a predominant band at approximately 57 kDa, indicating successful expression and high purity of the protein (**Figure 2A**, **Supplementary Figure S1**). The molecular size of the resulting proteins was further verified by WB analysis after purification on a Ni-NTA column (**Figure 2B**).

We then evaluated the binding of purified HLA-A2/KRAS G12V-CD3 BiTE to target cells (**Figure 2C, D**). Flow cytometry showed that the protein binds specifically to pancreatic cancer cells with KRAS G12V mutation in Capan-1 and CFPAC-1 as well as to human T cells (**Supplementary Figure S2A**). However, HLA-A2/KRAS G12V-CD3 BiTE showed no binding capacity to NUGC-4 (KRAS WT) (**Supplementary Figure S2B**).

### 3.2 Cell surface dual-modification with BiTE and DSPE-PEG-iRGD

Upon confirming the presence of receptors on CFPAC-1 and Capan-1 cell lines that specifically bind iRGD, including αv integrins (αvβ3, αvβ5) and neuropilin-1 (NRP-1) receptors (**Supplementary Figure S3**), we established a dual-modification system on the surface of T cells. As shown in **Figures 2E, F**, flow cytometry analysis quantitatively confirmed the successful anchorage of DSPE-PEG-iRGD on the T-cell surface, as evidenced by a substantial rightward shift in the fluorescence histogram and a significant increase in the mean fluorescence intensity (MFI) of FAM-labeled DSPE-PEG-iRGD in BiTE modified T cells compared to BiTE modified T cells control.

We first optimized the labeling conditions by incubating BiTE modified T cells with varying concentrations (0-40 μg) of FAM-labeled DSPE-PEG–iRGD. Flow cytometric analysis revealed a dose-dependent increase in the fluorescence intensity on T cells as the iRGD concentration increased (**Figure 2G**). When the amount of DSPE–PEG–iRGD reached 20 μg per 10^6^ cells, the fluorescence intensity reached a plateau, and the modification efficiency approached saturation (**Figure 2H**), indicating that this dosage was sufficient to achieve maximal surface modification of T cells. We further evaluated the *in vitro* stability of the dual-modified T cells over a 72-hour period. The results showed that at 24 hours post-modification, the surface fluorescence intensity remained above 70% of the initial level; by 72 hours, approximately 60% of the fluorescence signal was still retained (**Figure 2I**). These findings demonstrate that DSPE-PEG-iRGD exhibits favorable retention capacity on the T-cell membrane, providing a stability basis for the *in vivo* application of this modification strategy.

**Figure 3:**
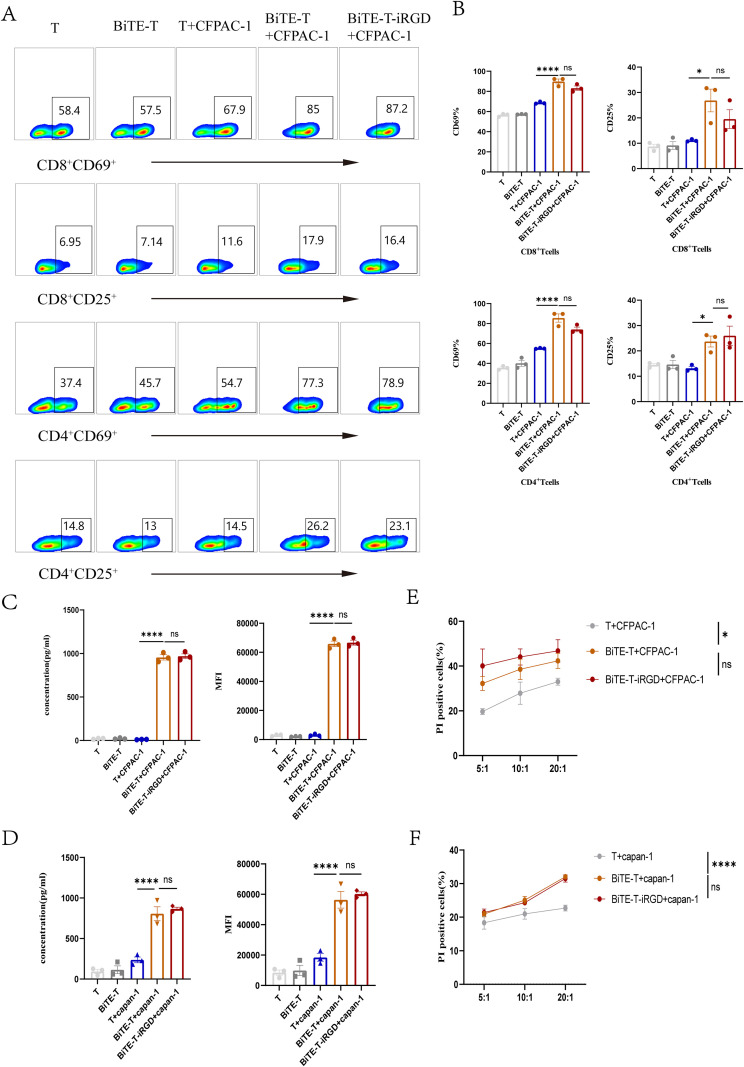
Influence of DSPE-PEG-iRGD modification on BiTE-T cells *in vitro*. **(A)** Phenotypes of cultured lymphocytes in different format were analyzed by flow cytometry. **(B)** The bar graphs showed a comparison of T cells from a representative individual on T cell phenotypic markers of different T cell subsets. **(C)** The bar graphs demonstrated the secretion of IFN-γ from T cells co-cultured with CFPAC-1. **(D)** The bar graphs demonstrated the secretion of IFN-γ from T cells co-cultured with Capan-1. **(E)** The cytotoxic reactivity of T cells was measured using CFSE/PI cytotoxicity assay, the target cell was CFPAC-1. **(F)** The cytotoxic reactivity of T cells was measured using CFSE/PI cytotoxicity assay, the target cell was Capan-1. Data are represented as mean ± s.e.m.; n = 3. *p < 0.05, ****p < 0.0001, ns, not significant.

Following the successful establishment of the modification system, we systematically evaluated its impact on the core functions of T cells. The dose of the HLA-A2/KRAS G12V-CD3 BiTE was determined with reference to that reported previously (14), we observed that in the presence of KRAS G12V-mutated tumor cells, the BiTE effectively activated T cells. This was evidenced by the upregulated surface expression of the early activation marker CD69 and the late activation marker CD25, along with increased secretion of IFN-γ and IL-2 (**Figures 3A–D**, **Supplementary Figure S5C**). Cytotoxicity assays further confirmed that the BiTE directed T cells to induce a stronger lytic effect against target cells across various effector-to-target (E: T) ratios (**Figures 3E, F**). Crucially, the introduction of DSPE-PEG-iRGD did not cause significant adverse effects on T cell phenotype, IFN-γ and IL-2 secretion, killing capacity, or its binding to BiTE. Furthermore, the CFSE/PI and CFSE dilution assays showed that even high concentrations of DSPE-PEG-iRGD did not affect T cell viability or proliferative capacity (**Figures 3A–F**, **Supplementary Figure S2A**, **Supplementary Figures S4A, B**, **Supplementary Figure S5**). These results collectively demonstrate the feasibility and stability of the iRGD modification approach and its excellent compatibility with BiTE function.

### 3.3 Dual modification facilitated T cells penetration in MCSs

To reflect the infiltration of different groups of T cells, we constructed three-dimensional multicellular sphere (MCS) using the pancreatic cancer cell line Capan-1.

**Figure 4:**
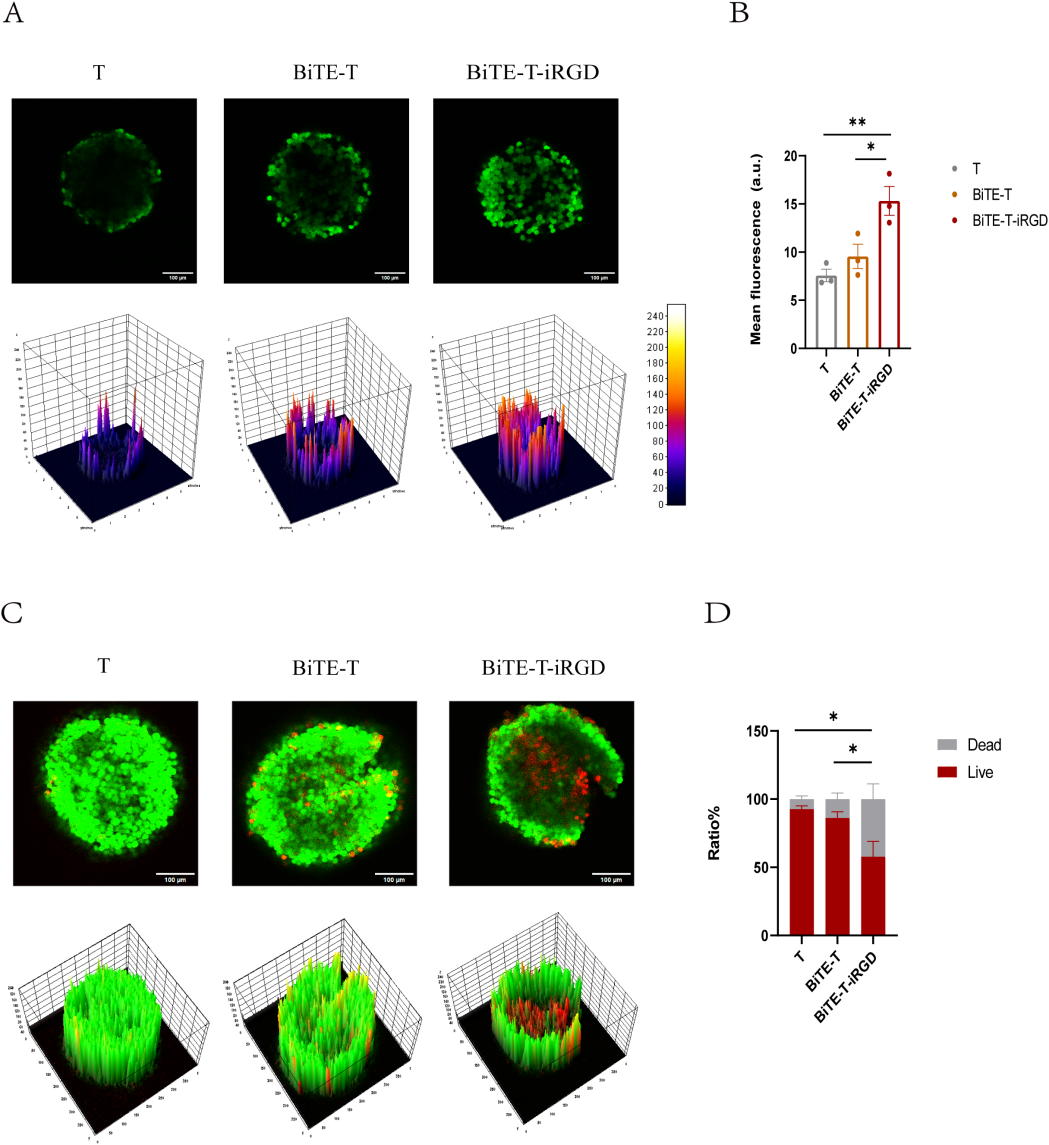
DSPE-PEG-iRGD modification improved the cytotoxicity of BiTE-T cells against capan-1tumor spheroids. **(A)** Confocal microscopy images (upper panel) and surface plot images (lower panel) showing penetration of CFSE-labeled T cells in MCSs. Magnification, ×200; scale bar, 100 μm. **(B)** Mean CFSE fluorescence intensity of MCSs in **(A)**. **(C)** Confocal microscopy images (upper panel) and surface display (lower panel) of live/dead viability assays in MCSs. Live cells were stained with calcein acetoxymethyl ester (AM) (green) and dead cells with PI (red); magnification, ×200; scale bar, 100 μm. **(D)** Live/dead cell quantification in MCSs. Data are represented as mean ± s.e.m.; n=3. *p < 0.05, **p < 0.01, ns, not significant.

First, we test the infiltration of T cells. Our study revealed that T cells alone, when unmodified, predominantly remained at the surface of the multicellular spheroids and only a weak fluorescence signal was detected in the confocal images. Although the HLA-A2/KRAS G12V-CD3 BiTE modification improved T cell penetration, it did not have a significant difference compared to unmodified T cells. However, when T cells were dual-modified with both BiTE and DSPE-PEG-iRGD, they exhibited enhanced penetration, reaching deeper into the spheroids. Remarkably, the fluorescence intensity consistently increased in the dual-modified T cell group (**Figures 4A, B**).

Then, we test the cytotoxicity of the infiltrating T lymphocytes. Different groups of T cells were separately cocultured with Capan-1 MCSs at an E/T ratio of 10:1 for 16 h. The integrity and diameter changes of tumor spheroids were monitored by microscopy. Representative images showed that T cells alone and HLA-A2/KRAS G12V-CD3 BITE-modified T cells exhibited limited inhibition of MCSs, while MCSs growth were significantly suppressed in the dual-modified group (**Supplementary Figure 6A–C**). We also used Calcein AM and PI solutions to examine the killing capacity of T cells in Capan-1 MCSs. Results indicated that tumor cells in the T alone were all mostly alive, BiTE led to a limited amount of cell death, whereas T cells with dual modification were found to be more effective in killing tumor cells (**Figures 4C, D**). These experiments demonstrated that iRGD modification could enhance the infiltration capacity of HLA-A2/KRAS G12V-CD3 BiTE for a stronger cytotoxic effect.

### 3.4 iRGD promoted BiTE modified T cells accumulation *in vivo*

**Figure 5:**
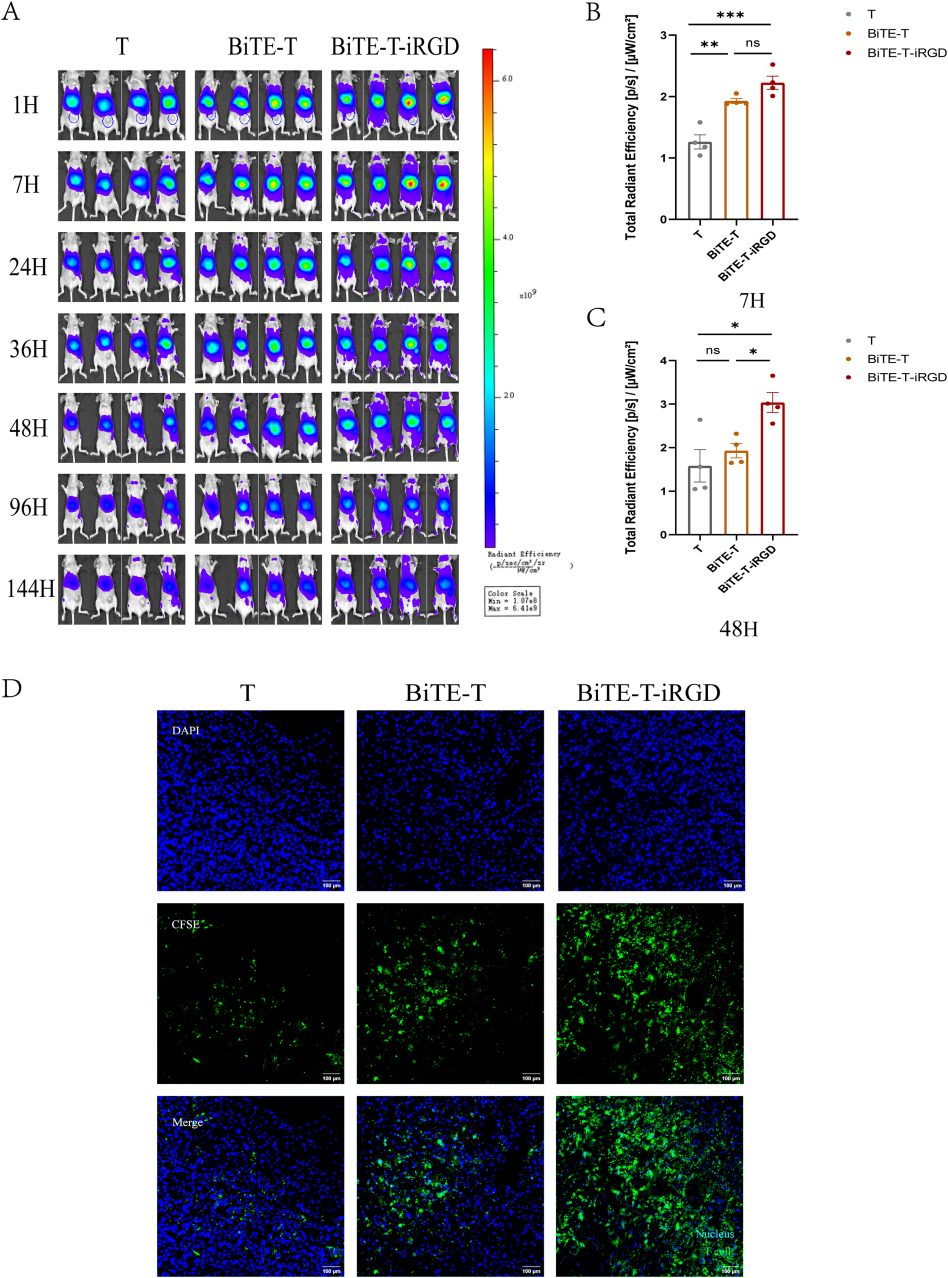
iRGD modification enhanced BiTE-T lymphocytes infiltration into tumor parenchyma in a systematic administration route. **(A)***In vivo* imaging of CFPAC-1 tumor-bearing mice at different times after intravenous injection of DiR-labeled T cells with different treatment. **(B)** Total radiant efficiency of different groups of T cells at 7h in tumors *in vivo*. **(C)** Total radiant efficiency of different groups of T cells at 48h in tumors *in vivo*. **(D)** Confocal imaging of frozen tumor sections at 24 h after injection. T cells were labeled with CFSE before intravenous injection; T cells, green; nucleus, blue. Scale bar, 100 μm. Data are represented as mean ± s.e.m. *p < 0.05, **p < 0.01, ***p < 0.001, ns, not significant.

We evaluated the infiltration and accumulation of dual-modified T cells in a CFPAC-1 pancreatic subcutaneous tumor model. Near-infrared imaging demonstrated that all of the groups had different degrees of lymphocyte accumulation in tumors post intravenous injection (**Figure 5A**). Although there was a significant difference in fluorescence intensity at 7 h after cell transfusion due to HLA-A2/KRAS G12V-CD3 BITE compared to the T group alone (**Figure 5B**), it was not statistically significant at 48 h. However, dual-modified T cells exhibited strong signal in tumors 48 h after cell transfusion (**Figure 5C**). The fluorescence signal of the resected tumor even was the strongest compared to the other two groups after a week (**Supplementary Figures S7A, B**). These results showed that dual-modified T cells effectively accumulated in tumors and exerted more sustained anti-tumor effects.

We then performed immunofluorescence analysis to further assess T cell penetration (**Figure 5D**). Immunofluorescence analysis revealed that untreated T cells was scattered in tumor tissues after 24 h of intravenous infusion. The presence of BiTE led to a notable accumulation and increase of T cells in tumor tissues. HLA-A2/KRAS G12V-CD3 modification endowed T cells with infiltration capacity in tumor tissues *in vivo*. However, its accumulation area in tumor tissues was limited. Notably, the accumulation and infiltration capacity of T cells were more significantly improved after iRGD modification, with a significantly larger number of T cells and an enlarged aggregation area.

### 3.5 Dual-modified T cells mediated superior antitumor effect

**Figure 6:**
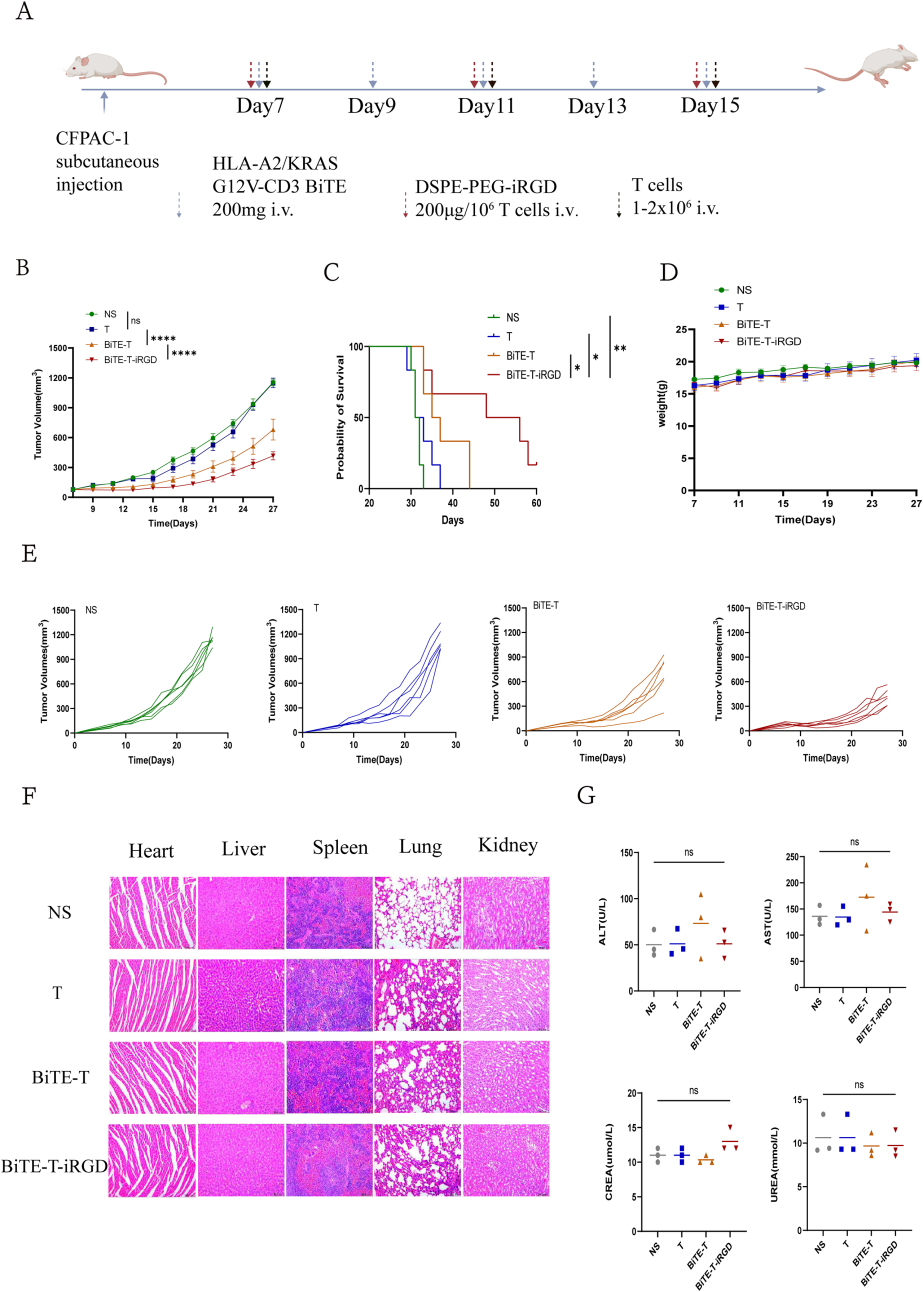
Dual-modification T cells effectively controlled tumor growth. **(A)** Schematic diagram of administration route of subcutaneous pancreatic mouse models (n=6). Created with BioRender.com. **(B)** Average tumor-growth curves in CFPAC-1 subcutaneous mouse model (n=6). **(C)** Survival curves of different groups for 60 days (n=6). **(D)** Average weight of different groups for 27 days (n=6). **(E)** Tumor-growth curves of each mouse in different group (n=6). **(F)** Hematoxylin-eosin staining of heart, liver, spleen, lung, and kidney in CFPAC-1 subcutaneous mouse model on 2 weeks after the last treatment (n=3). The scale bars were 100 μm. **(G)** Blood biochemistry and hematology data on 2 weeks after the last treatment (n = 3). ALT, glutamic-pyruvic transaminase; AST, aspartate aminotransferase; CREA, creatinine; Data are represented as mean ± s.e.m. *p < 0.05, **p < 0.01, ****p < 0.0001, ns, not significant.

To assess the effectiveness of KRAS G12V high targeted-infiltration T cells against tumors *in vivo*, we first established CFPAC-1 pancreatic cancer models. After the tumor volume reached around 80~100 mm^3^, 100 μg HLA-A2/KRAS G12V-CD3 BiTE was given intravenously every 2 days, 1×10^7^ T cells and 200 μg DSPE-PEG-iRGD every 4 days (**Figure 6A**). By following tumor growth, we found that tumors in NS and T cells groups grew promptly, while 100 μg HLA-A2/KRAS G12V-CD3 BiTE modified activated T cells caused significantly tumor regression (**Figures 6B, E**). While these treatments prolonged survival to a certain extent, there was no statistical difference when comparing the results of these two groups with the T cells alone group. With the modification of DSPE-PEG-iRGD, both tumor inhibition and survival had further improved (**Figure 6C**). Conversely, mice from all other treatment groups succumbed within 44 days. Furthermore, no significant difference in the body weight were observed (**Figure 6D**). As reported in the literature regarding the function of iRGD (13, 16), We conclude that iRGD modification breaks through the limitation of the dense matrix surrounding tumors leading to insufficient infiltration of KRAS-targeted T cells, and extremely promotes the efficacy of HLA-A2/KRAS G12V-CD3 BiTE.

### 3.6 Biosafety of dual-modified T cells

Cellular immunotherapy may pose potential risks. In order to assess the toxicity of dual-modified T cells, H&E-stained images of major organs such as the heart, liver, spleen, lungs, and kidneys were collected for histopathological examination after the last treatment showed no histological abnormalities in all groups (**Figure 6F**). In addition, we analyzed serum biochemical markers including in alanine aminotransferase (ALT), aspartate aminotransferase (AST), creatinine (CREA) and UREA in treated mice, revealing no significant differences in each group (**Figure 6G**). Our results show no signs of severe toxicity. These findings affirmed the biosafety and efficacy of dual-modified-T therapy *in vivo*.

## 4 Discussion

The rates of morbidity and mortality of pancreatic cancer are increasing every year. In the past, KRAS proteins have been considered non-druggable due to their smooth structure and lack of good sites for drug targeting, as well as its high affinity for GTP (17). With the emergence of KRAS G12C covalent inhibitors and the rise of adoptive cell therapy (ACT) strategies (18–22), a large number of new KRAS-targeted investigational drugs are entering the clinical phase. Presentation of antigenic peptides to the cell surface using the major histocompatibility complex (MHC) has been a cornerstone of immunotherapy (23). HLA-A2/KRAS G12V-CD3 BiTE is an immuno-oncology therapy targeting KRAS G12V for the treatment of solid tumors. We have demonstrated that it has good anti-tumor value (14). Previously, our laboratory combined iRGD and Distearoylphosphoehanolamine-polyethylene glycol Maleimide, (DSPE-PEG-mal) to form a lipid insert that guided the drug-carrying system and cells to penetrate the vessel wall and enter the interior of solid tumors (15, 24). In this study, we achieved potentiation of HLA-A2/KRAS G12V-CD3 BiTE, via modification of a tumor-penetrating peptide that induce T-cell responses. Potent homing of T cells into the tumor tissue was achieved by the penetrating effect of iRGD, BiTE further activated these T cells in the tumor and facilitated deep infiltration into the tumor center to enhance the anti-tumor efficacy of immunotherapy.

Bispecific antibody can specifically bind antibodies to two different antigens and has potential in tumor immunotherapy due to its bifunctionality. However, the therapeutic efficiency of bispecific antibody depends on the level of infiltration of immune cells and the tumor microenvironment. There are multiple possibilities to limit the infiltration of T lymphocytes into the tumor parenchyma after entering the blood circulation. T cell ‘homing defects’ may be a major cause to ineffective immunotherapy outcomes (25). Expression of adhesion proteins ICAM-1 and VCAM-1 is downregulated in vascular-related endothelial cells, preventing normal rolling and adhesion of T cells to blood vessels (26). As early as 2000, Hanahan and Weinberg noted that neovascularization is intricately linked to tumor growth (27), and they went further to point out the low immunocompetence of T cells in the tumor microenvironment (28). T cells infiltrating the tumor parenchyma are influenced by suppressor immune cells within the tumor microenvironment, including myeloid-derived suppressor cells (MDSCs), M2-type tumor-associated macrophages (TAMs), and regulatory T cells (Tregs), as well as cytokines (IL-10, TGF-β), negative co-stimulatory molecules, and hypoxic conditions, all of which modulate the anti-tumor efficacy of T cells (29, 30). Numerous studies are actively investigating strategies to enhance T-cell infiltration into tumors (31, 32). Notably, immunotherapy, which are increasingly being adopted in the clinic, are in high demand to tackle this challenge. This is critical given that only a small fraction, less than 1% (13), of the administered T-cells successfully infiltrate the tumor tissue (33).

Combining the structure of bispecific antibodies, Zhou et al. designed and successfully constructed the fusion protein iRGD-antiCD3 (34). iRGD-anti-CD3 can bind circulating T cells via CD3 single-chain antibody fragments, and under the guidance of iRGD, the bound T cells can be targeted locally to the tumor and further infiltrate deeper into the tissue. Here we provided another efficient strategy by functionalizing T cells with the tumor-penetrating peptide iRGD in combination with bsAb. The iRGD-modified BiTE-directed T-cell therapeutic approach endows effector cells with dual capabilities: precise targeting of specific antigens and potent penetrating power. IRGD can bind to integrins and increase T cell accumulate in tumor vessels (35, 36) and then bind to the NRP-1 receptor that is highly expressed on the surface of tumor cells and tumor vascular endothelial cells (16, 37).

Our previous studies have demonstrated that HLA-A2/KRAS G12V-CD3 BiTE delayed tumor growth. However, the weak binding strength of HLA-A2/KRAS G12V-CD3 BiTE to T cells may prevent T cells from being functionalized. Because T cells can produce cytotoxic effects only if they bind to both tumor cells and BiTE. Moreover, the efficacy of the HLA-A2/KRAS G12V-CD3 BiTE is inherently restricted to patients whose tumors express the HLA-A2 allele, as this molecule is essential for presenting the KRAS G12V neoantigen to BiTE-engaged T cells. This fundamental characteristic confines the current therapeutic application to the HLA-A2-positive patient population. However, a substantial proportion of patients who are HLA-A2-negative cannot benefit from this specific therapeutic strategy. To overcome this limitation and broaden the applicability of immunotherapy, several strategic avenues can be pursued. First, the core technological platform can be leveraged to develop parallel BiTE molecules that recognize the KRAS G12V mutant peptide presented by other common HLA subtype, thereby extending coverage to a wider patient population. Second, HLA-independent cellular immunotherapy approaches can be explored, such as T cells engineered with transgenic T-cell receptors (TCRs) specific for the KRAS G12V neoantigen, or therapies based on natural killer (NK) cells. Furthermore, combination strategies employing epigenetic modulators (DNA methyltransferase inhibitors) could be investigated to upregulate the overall expression of HLA class I molecules on tumor cell surfaces (38). This approach may sensitize a subset of tumors that are initially immunologically “silent” to HLA-restricted therapies.

Pancreatic cancer is a low-immunogenic, highly immunosuppressive tumor type for which immunotherapy is extremely limited in the clinic. Pancreatic cancer is distinguished from other solid tumors by its mesenchymal components, which constitute over 80% of the tumor volume. This includes a rich extracellular matrix, vasculature, and tumor-associated fibroblasts (CAFs). These components encircle the tumor parenchyma, creating a stromal barrier that lead to the progression of pancreatic cancer and the inability of therapeutic agents to efficiently enter the tumor nests (39). We consider that the efficacy of HLA-A2/KRAS G12V-CD3 BiTE is also limited by the influence of the microenvironment. In this study, we used a lipid insert with iRGD to induce T-cell infiltration into the tumor, resulting in improved CD3 BiTE treatment outcomes. A significant advantage of this strategy is that T cells functionalized to bind specifically to the antibody are more effectively recruited to the target area. Concurrently, T cells that cannot bind to the antibody also accumulate at the tumor site due to iRGD modification. This dual-action approach can be likened to the deployment of two catalysts for T cell infiltration.

In summary, we demonstrates that combining the iRGD-mediated penetration strategy with BiTE technology targeting the KRAS G12V neoantigen synergistically enhances T cell infiltration. The dual modification of T cells with both iRGD and BiTE resulted in significantly improved penetration capability and cytotoxic activity in both multicellular spheroids and xenograft tumor models. This work provides proof-of-concept for integrating iRGD-based penetration technology with BiTE therapy directed against specific tumor neoantigens, highlighting the unique value of this combinatorial approach in addressing the dual challenges of infiltration and targeting in solid tumors.

## Data Availability

The raw data supporting the conclusions of this article will be made available by the authors, without undue reservation.

## Supplementary material

Supplementary Figure 1 Purity was confirmed by 12% SDS-PAGE.

Supplementary Figure 2 **(A)** Flow cytometry results showing bindings of HLA-A2/KRAS G12V-CD3 BiTE to PBMC. **(B)** Flow cytometry results showing bindings of HLA-A2/KRAS G12V-CD3 BiTE to NUGC-4.

Supplementary Figure 3 Histograms showing the expression of αvβ3, αvβ5 and NRP-1 on CFPAC-1 and Capan-1.

Supplementary Figure 4 **(A)** T cell viability before and after modification with varying concentrations of iRGD. **(B)** Proliferation of T cells was assessed by CFSE dilution.

Supplementary Figure 5 **(A)** Phenotypes of cultured lymphocytes in different format with Capan-1 were analyzed by flow cytometry. **(B)** The bar graphs showed a comparison of T cells from a representative individual on T cell phenotypic markers of different T cell subsets. **(C)** The bar graphs demonstrated the secretion of IL-2 from T cells co-cultured with CFPAC-1. Data are represented as mean ± s.e.m.; n=3. ns, not significant.

Supplementary Figure 6 **(A)** Growth inhibition assay in Capan-1 MCSs (n=6). **(B, C)** Representative images of MCS in different groups. scale bar, 100 μm. Data are represented as mean ± s.e.m. *p < 0.05, **p < 0.01, ***p < 0.001, ****p < 0.0001, ns, not signific

Supplementary Figure 7 **(A)** Ex vivo images of tumor, liver, and spleen at 168h after intravenous injection. **(B)** Total radiant efficiency of different groups of T cells at 168h in tumors *in vivo*. Data are represented as mean ± s.e.m. *p < 0.05. ns, not significant.
