# Correction: Tumor-penetrating peptide boosts bispecific T-cell engager antitumor efficacy for the pancreatic cancer

**DOI:** 10.3389/fimmu.2025.1761643

**Published:** 2025-12-12

**Authors:** Frontiers Production Office

**Affiliations:** Frontiers Media SA, Lausanne, Switzerland

**Keywords:** KRAS G12V, bispecific T-cell engager (BiTE), IRGD, pancreatic cancer, T-cell infiltration

The Editor Christian Klein’s affiliation “Ludwig Maximilian University of Munich, Germany” was erroneously given as “Roche Innovation Center Zurich, Switzerland”.

The original version of this article has been updated.

