# Correction: Tumor-penetrating peptide boosts bispecific T-cell engager antitumor efficacy for the pancreatic cancer

**DOI:** 10.3389/fimmu.2025.1772783

**Published:** 2026-01-07

**Authors:** Lu Zou, Jingjing Chen, Xinyuan Bai, Yingxin Wang, Changchang Lu, Qiaoli Wang, Subiyinuer Tuerhong, Mengzhu Li, Qinghua Zheng, Fanyan Meng, Juan Du

**Affiliations:** 1 Department of Oncology, Nanjing Drum Tower Hospital, Clinical College of Nanjing Drum Tower Hospital, Nanjing University of Chinese Medicine, Nanjing, China; 2 The Comprehensive Cancer Center of Nanjing Drum Tower Hospital, Affiliated Hospital of Medical School, Nanjing University, Nanjing, China; 3 Department of Laboratory Medicine, Nanjing Drum Tower Hospital, Clinical College of Nanjing Medical University, Nanjing, China; 4 Institute of Translational Medicine, Zhejiang University, Hangzhou, China; 5 State Key Laboratory of Analytical Chemistry for Life Sciences, School of Chemistry and Chemical Engineering, Nanjing University, Nanjing, China

**Keywords:** KRAS G12V, bispecific T-cell engager (BiTE), IRGD, pancreatic cancer, T-cell infiltration

An incorrect **Funding** statement was provided. The correct funder is “the National Natural Science Foundation of China (No. 82373280), Key Project supported by Medical Science and technology development Foundation, Nanjing Department of Health (ZKX24021 and YKK24072), the provincial key medical disciplines during the “14th Five-Year Plan” period (ZDXK202233) and 2024 Annual Medical Research Projects of the Jiangsu Provincial Health Commission (K2024038)”.

The original version of this article has been updated.

