# Supplementary figures and images for: Tumor-penetrating peptide boosts bispecific T-cell engager antitumor efficacy for the pancreatic cancer
Source: Front Immunol. 2025 Dec 4;16:1693755. doi: 10.3389/fimmu.2025.1693755 (PMC12745974; doi:10.3389/fimmu.2025.1693755)

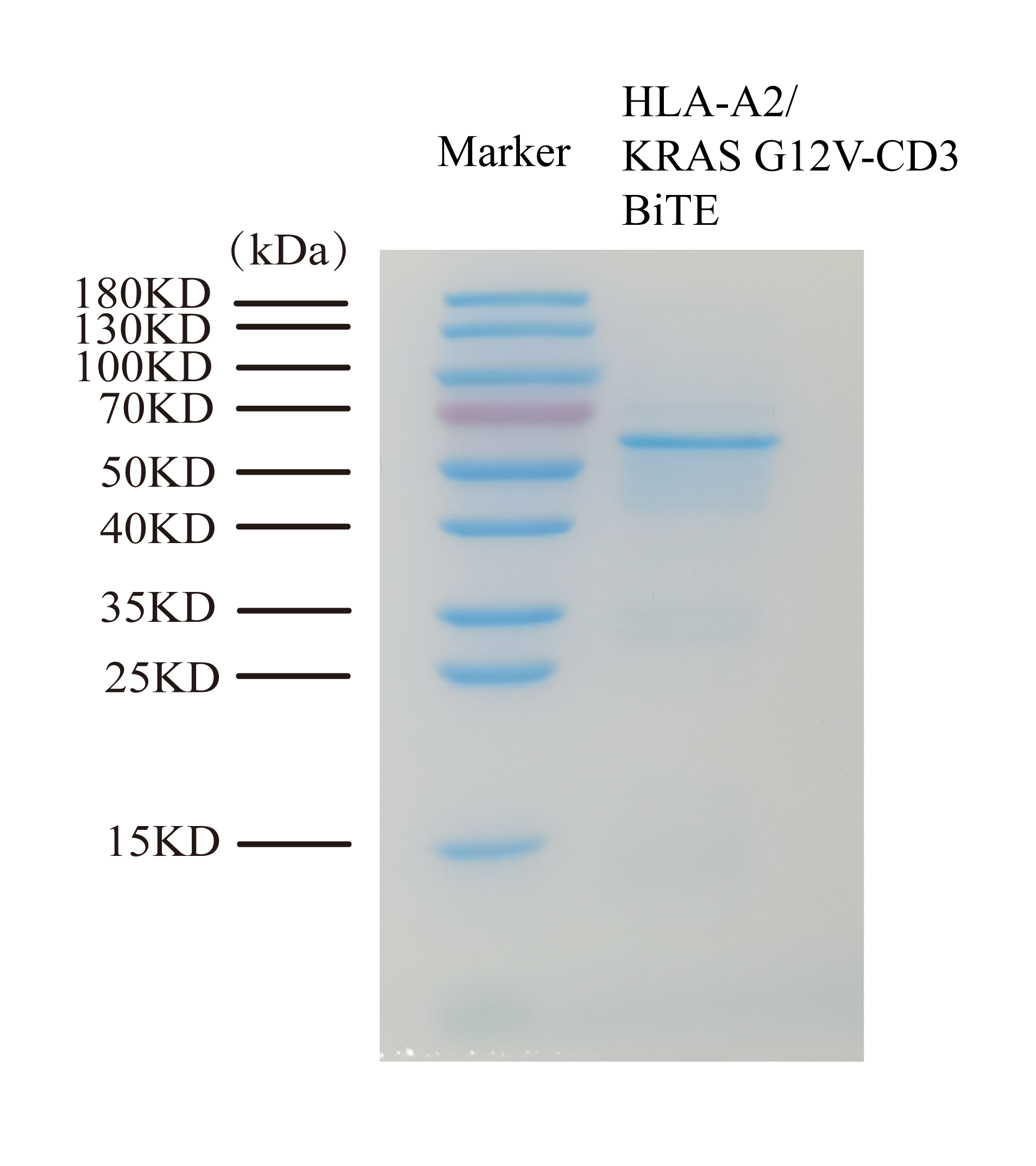

Supplement: Supplementary Figure 1 — Purity was confirmed by 12% SDS-PAGE. [file Image1.tif]

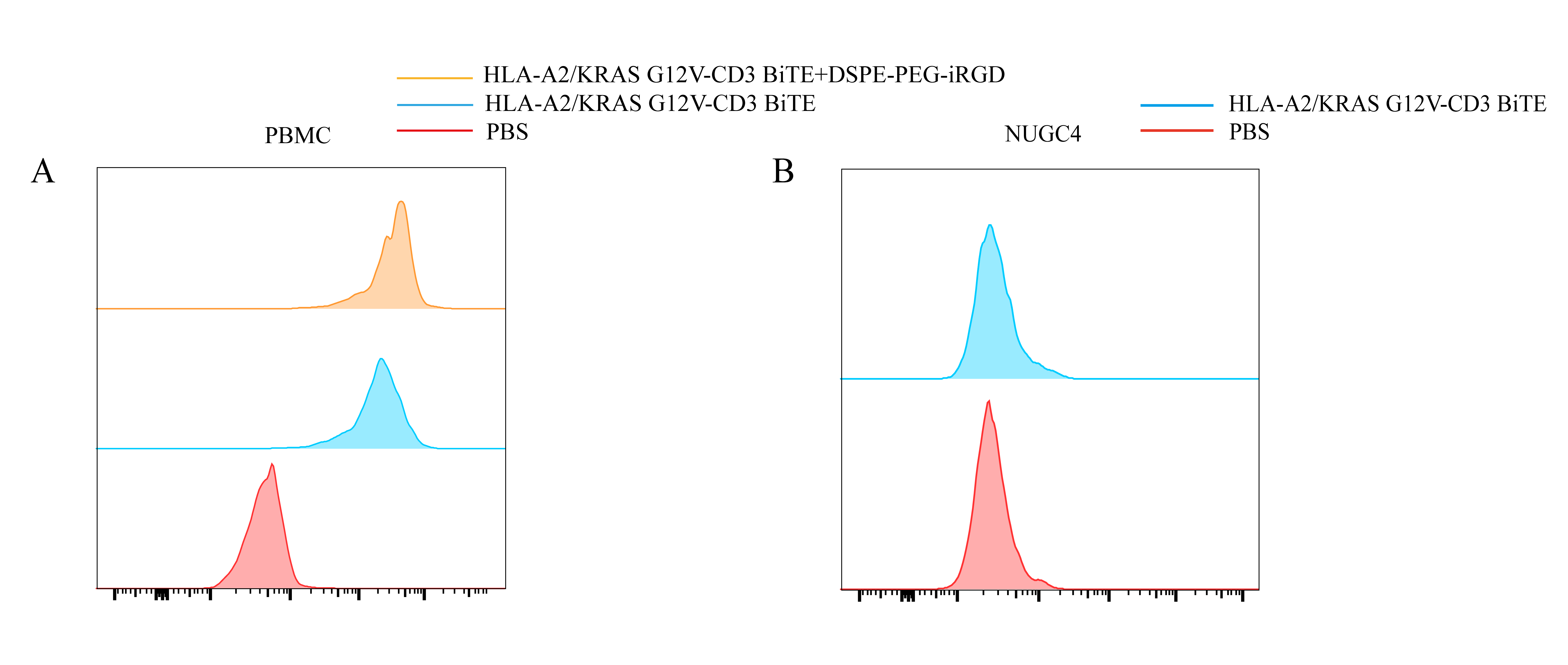

Supplement: Supplementary Figure 2 — (A) Flow cytometry results showing bindings of HLA-A2/KRAS G12V-CD3 BiTE to PBMC. (B) Flow cytometry results showing bindings of HLA-A2/KRAS G12V-CD3 BiTE to NUGC-4. [file Image2.tif]

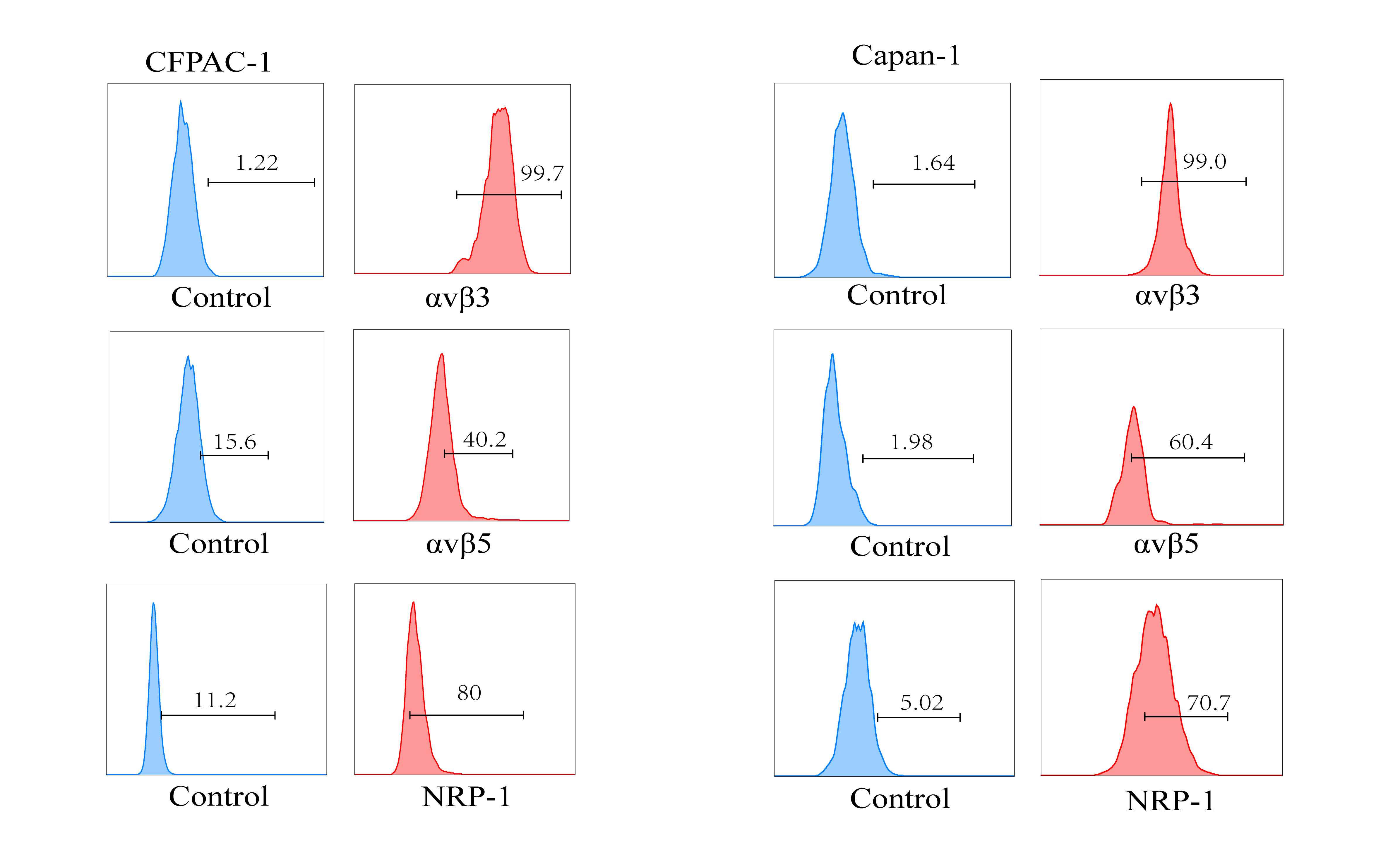

Supplement: Supplementary Figure 3 — Histograms showing the expression of αvβ3, αvβ5 and NRP-1 on CFPAC-1 and Capan-1. [file Image3.tif]

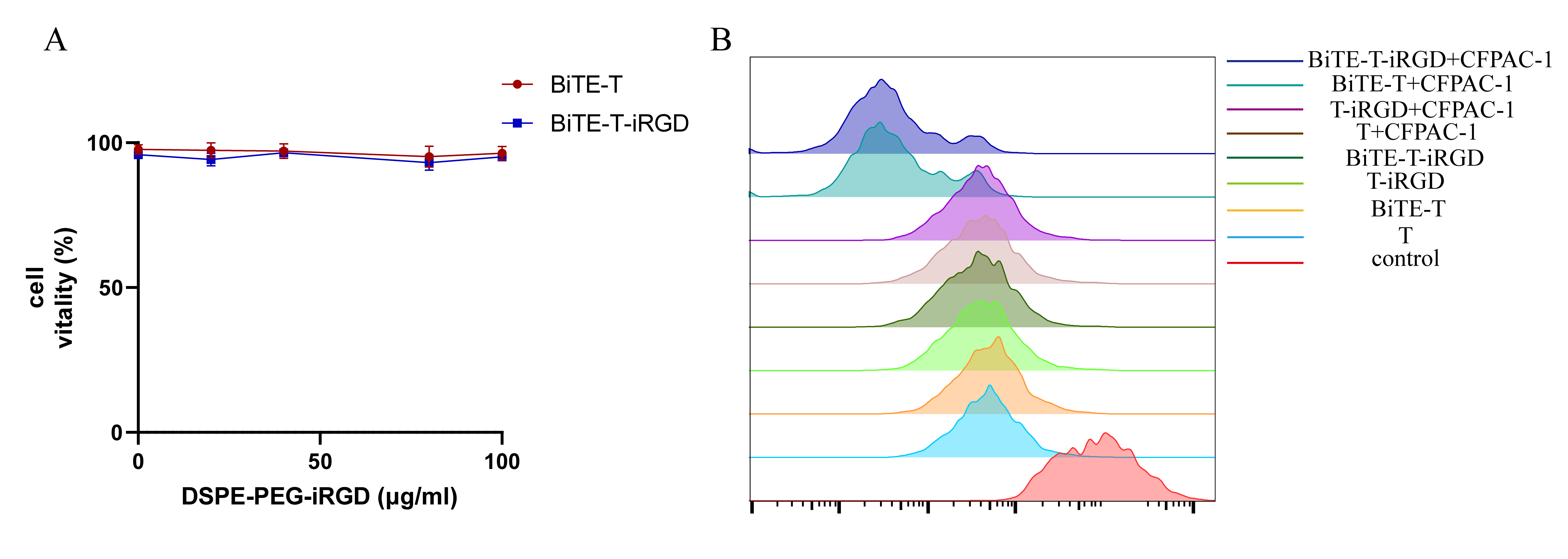

Supplement: Supplementary Figure 4 — (A) T cell viability before and after modification with varying concentrations of iRGD. (B) Proliferation of T cells was assessed by CFSE dilution. [file Image4.tif]

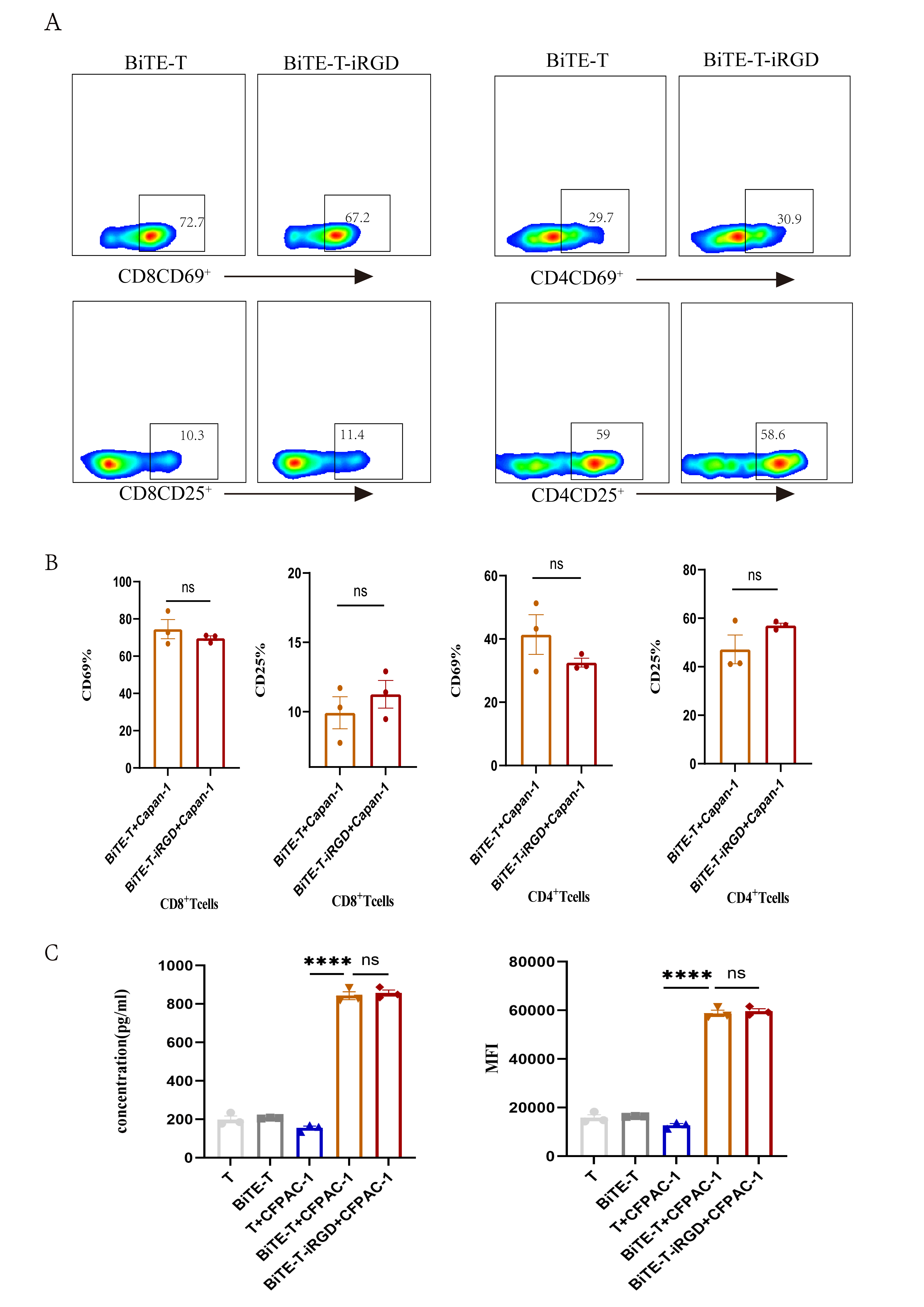

Supplement: Supplementary Figure 5 — (A) Phenotypes of cultured lymphocytes in different format with Capan-1 were analyzed by flow cytometry. (B) The bar graphs showed a comparison of T cells from a representative individual on T cell phenotypic markers of different T cell subsets. (C) The bar graphs demonstrated the secretion of IL-2 from T cells co-cultured with CFPAC-1. Data are represented as mean ± s.e.m.; n=3. ns, not significant. [file Image5.tif]

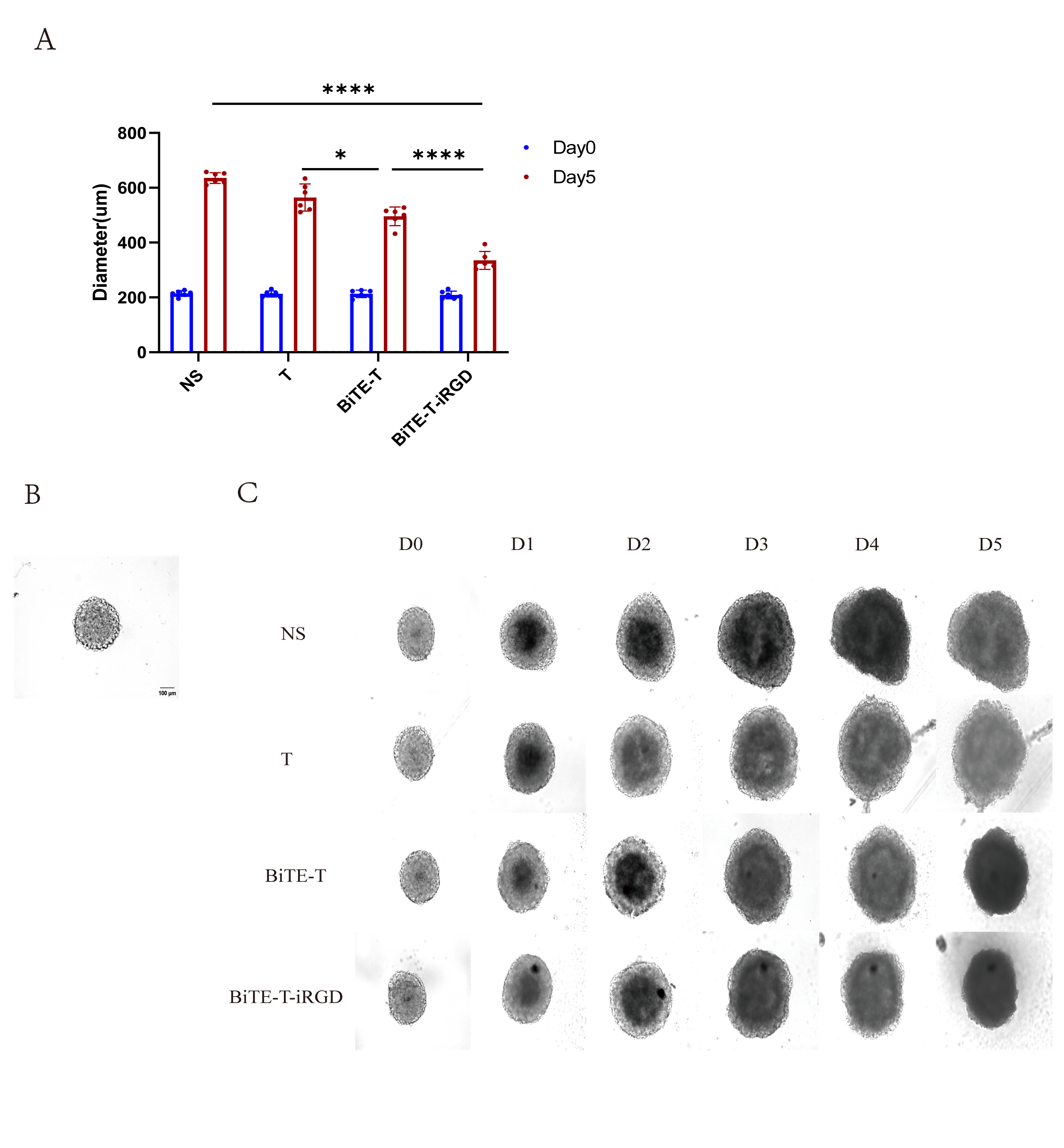

Supplement: Supplementary Figure 6 — (A) Growth inhibition assay in Capan-1 MCSs (n=6). (B, C) Representative images of MCS in different groups. scale bar, 100 μm. Data are represented as mean ± s.e.m. *p < 0.05, **p < 0.01, ***p < 0.001, ****p < 0.0001, ns, not signific [file Image6.tif]

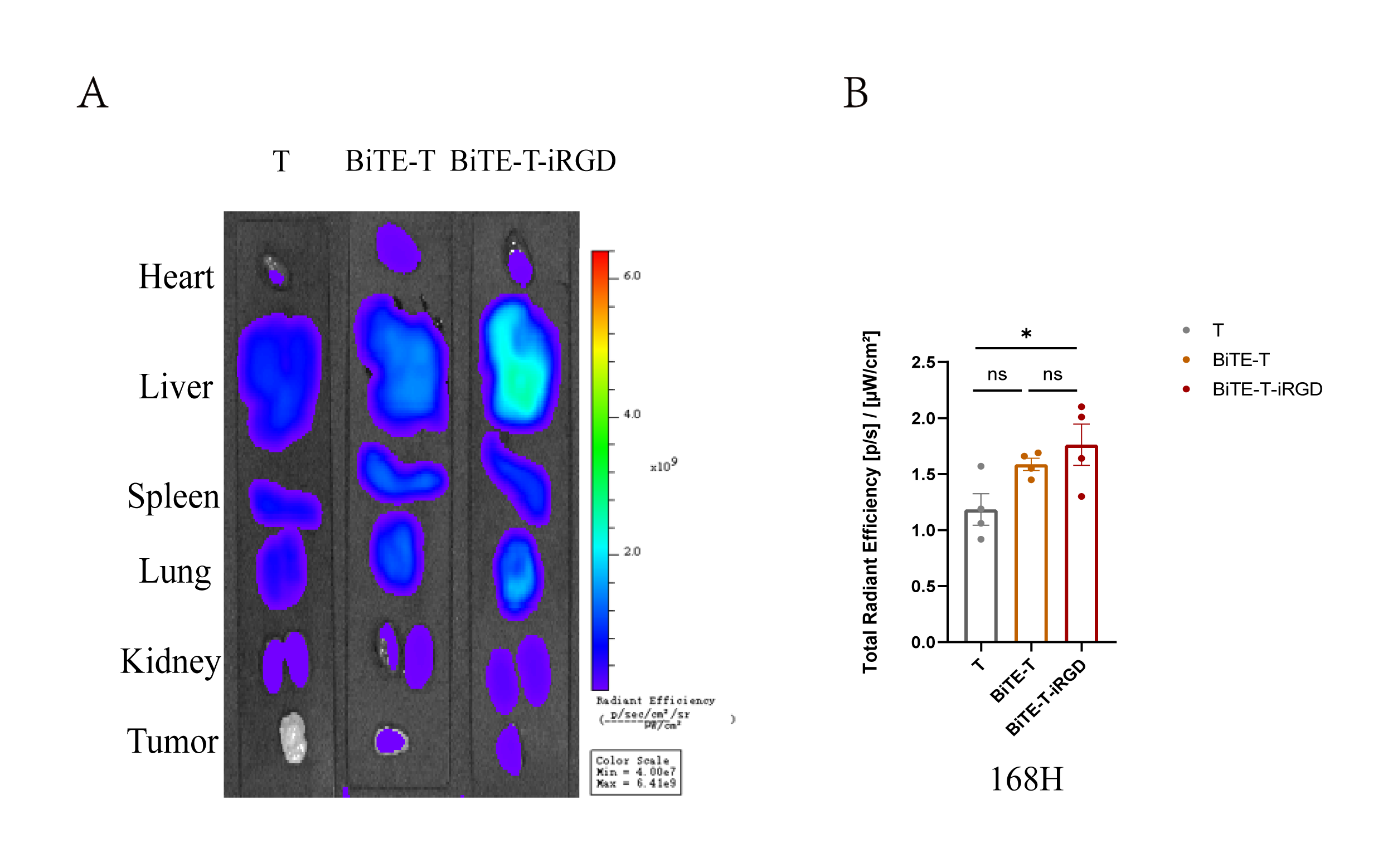

Supplement: Supplementary Figure 7 — (A) Ex vivo images of tumor, liver, and spleen at 168h after intravenous injection. (B) Total radiant efficiency of different groups of T cells at 168h in tumors in vivo. Data are represented as mean ± s.e.m. *p < 0.05. ns, not significant. [file Image7.tif]
